# Text Messages to Curb Sugar-Sweetened Beverage Consumption among Pregnant Women and Mothers: A Mobile Health Randomized Controlled Trial

**DOI:** 10.3390/nu13124367

**Published:** 2021-12-05

**Authors:** Jennifer A. Woo Baidal, Kelsey Nichols, Nalini Charles, Lauren Chernick, Ngoc Duong, Morgan A. Finkel, Jennifer Falbe, Linda Valeri

**Affiliations:** 1 Department of Pediatrics, Columbia University Irving Medical Center, NewYork-Presbyterian Morgan Stanley Children’s Hospital, New York, NY 10032, USA; Kelsey.m.m.nichols@gmail.com (K.N.); nqd2000@cumc.columbia.edu (N.D.); maf2260@cumc.columbia.edu (M.A.F.); 2 New York Presbyterian Hospital Special Supplemental Nutrition Program for Women, Infants and Children, 622 W. 168th Street, New York, NY 10032, USA; nac9026@nyp.org; 3 Department of Emergency Medicine, Columbia University Irving Medical Center, NewYork-Presbyterian Morgan Stanley Children’s Hospital, New York, NY 10032, USA; lc2243@cumc.columbia.edu; 4 Department of Biostatistics, Columbia University Mailman School of Public Health, New York, NY 10032, USA; lv2424@cumc.columbia.edu; 5 Human Development and Family Studies Program, Department of Human Ecology, University of California, Davis, CA 95616, USA; jfalbe@ucdavis.edu

**Keywords:** sugar-sweetened beverage, obesity, pregnant women and mothers, mobile health, randomized controlled trial, graphic beverage health warning labels, beverage sugar content information

## Abstract

Racial, ethnic, and socioeconomic disparities in childhood obesity in the United States (U.S.) originate in early life. Maternal sugar-sweetened beverage (SSB) consumption is an early life risk factor for later offspring obesity. The goal of this study was to test the effects of policy-relevant messages delivered by text messages mobile devices (mHealth) on maternal SSB consumption. In this three-arm 1-month randomized controlled trial (RCT), pregnant women or mothers of infants in predominantly Hispanic/Latino New York City neighborhoods were randomized to receive one of three text message sets: graphic beverage health warning labels, beverage sugar content information, or attention control. The main outcome was change in maternal self-reporting of average daily SSB consumption from baseline to one month. Among 262 participants, maternal SSB consumption declined over the 1-month period in all three arms. No intervention effect was detected in primary analyses. In sensitivity analyses accounting for outliers, graphic health warning labels reduced maternal SSB consumption by 28 kcal daily (95% CI: −56, −1). In this mHealth RCT among pregnant women and mothers of infants, graphic health warning labels and beverage sugar content information did not reduce maternal SSB consumption.

## 1. Introduction

Among children under the age of 24 months in the United States (U.S.), high infant weight-for-length prevalence among Hispanic/Latino children (11.3%) and non-Hispanic Black children (10.2%) is higher than non-Hispanic White (8.8%) counterparts [1,2,3,4,5]. Childhood obesity risk factors exist during the first 1000 days—gestation through the age of 2 years—and are more prevalent among low socioeconomic groups and racial/ethnic minorities [6,7,8,9,10,11]. Racist practices and policies that promote segregation and discrimination, inequitable access to healthy, affordable foods, and targeted marketing of calorie-dense, nutrient-poor foods and beverages perpetuate racial/ethnic disparities in obesity risk factors starting early in life [12,13,14]. 

Maternal sugar-sweetened beverage (SSB) consumption in pregnancy and infant SSB consumption in infancy are risk factors for later childhood overweight/obesity [15,16] and other adverse child health consequences [17,18]. SSB consumption in the first 1000 days is relatively common, with U.S. Hispanic/Latino populations among those with the highest prevalence of consumption [15,19,20,21,22]. Maternal beverage attitudes and consumption have been linked to offspring beverage consumption [22,23]. Thus, interventions to curb SSB consumption and promote healthy beverage intake in low-income and Hispanic/Latino adults with children may help reduce maternal and infant SSB consumption, yet SSB-specific interventions in this life course period are lacking. In our prior formative qualitative research, pregnant women and mothers of infants identified graphic health warning labels and beverage-specific information on sugar content as motivating towards SSB avoidance [24]. Other SSB-reduction trials among parents of children have studied beverage selection responses to message frames at a single point in time, but real-world interventions measuring beverage consumption outcomes for parents and infants are lacking. Low-income households and racial/ethnic minorities have rapidly adopted mobile technology [25], thus mobile technology-based health interventions (mHealth) may be an effective method for delivering messages in real-world settings to support a reduction in SSB consumption among pregnant women and mothers of infants in populations disproportionately burdened by obesity. 

The overall goal of the proposed study is to test policy-relevant text messages delivered by mHealth to reduce SSB consumption during the first 1000 days among mothers in low-income households living in predominantly Hispanic/Latino neighborhoods. Intervention messages were framed as either (1) graphic health warning labels focused on negative health impacts of sugar consumption or (2) beverage sugar content information to support parental knowledge and choice. We hypothesized that adults in each intervention arm would have greater reductions in habitual SSB consumption compared to the control group. Secondarily, we hypothesized that infants in each intervention arm would have reduced likelihood of consuming SSBs at follow-up compared to infants in the control group.

## 2. Materials and Methods

### 2.1. Trial Design 

We performed a parallel, randomized control trial of messaging to reduce SSB consumption delivered by mHealth among pregnant women and parents of infants age < 24 months (hereafter termed mothers). We randomized with a 1:1 allocation ratio to 1 of 3 arms: (1) unhealthy beverage avoidance messages framed as graphic health warnings, (2) messages framed as information for parents on beverage-specific sugar content, and (3) attention control (infant safety information). The main outcome was change in maternal consumption of SSB over one month. 

Recruitment was unexpectedly divided into two phases because of COVID-19. In Phase 1, we recruited families from a multi-site special supplemental nutrition program for women, infants, and children (WIC) practice in New York City with a predominantly Hispanic/Latino population. Because of COVID-19 and cessation of all in-person WIC visits, we paused recruitment on 13 March 2020. In Phase 2, we recruited from well-child visits in a multi-site ambulatory care network (ACN) practice at an academic health care center in New York City serving a population similar to WIC, regardless of WIC enrollment status. We converted all study visits from in-person to virtual via computer-assisted telephone visits or by video.

### 2.2. Participants and Setting

In Phase 1, eligibility included the following: age ≥ 18 years, pregnant woman or legal caretaker of infant age < 24 months, and enrollment in WIC. Study staff performed on-site recruitment and obtained written consent at the two WIC sites. 

In Phase 2, eligibility included the following: age ≥ 18 years and legal caretaker of infant age < 24 months with a well-child visit in the NYPH-Ambulatory Care Network (ACN) at Columbia University Irving Medical Center. After a 2-week opt-out waiting period subsequent to study information mailings, study staff contacted potentially eligible families by telephone to establish eligibility and obtain written electronic consent.

For Phases 1 and 2, inclusion criteria were the ability to respond to questions in English or Spanish, daily use of a mobile device with iOS or Android platforms, and consent to receive text messages by mHealth. Exclusion criteria were chronic medical conditions impacting maternal or infant nutrition and prior enrollment of a household member in this trial. Enrolled participants completed a baseline survey and mHealth system orientation before randomization. Study staff who were fluent in English and/or Spanish completed research visits. All consent, survey materials, and mobile messages were available in English and Spanish. The Columbia University Irving Medical Center Institutional Review Board approved all study procedures. This RCT was registered on Clinicaltrials.gov (NCT04238585) on 16 January 2020 as “BabyQ’s: Randomized Controlled Trial of Health Messaging in Pregnancy and Infancy”.

### 2.3. Interventions

The Theory of Planned Behavior and multi-level framework of influences underpinned the intervention development [26,27]. Intervention development included the following: (1) a systematic review of the literature [28,29]; (2) a review of the existing interventions, policies, and campaigns to reduce SSB consumption; and (3) mixed methods formative research [22,24]. Intervention messages were refined through an iterative process using key expert and parental feedback and refinement. 

Participants received one of three message types for a 1-month period. Message format and frequency were similar across all arms (Table 1; full set available upon request). Personalized messages with a text prompt and a link to an illustrative message were delivered by mHealth three times weekly for the 1-month intervention. Intervention messages focused on healthy beverage goals to promote avoidance of SSB consumption, limit juice intake, and drink at least eight glasses per day of water. Each intervention arm received eight SSB consumption messages, as well as one juice, one water, three motivational, and one welcome message. The main intervention outcome was reduction of SSB consumption among pregnant women and parents of infants.

1. Graphic health warning labels (Ix 1)

Participants in Ix 1 received messages illustrating adverse health impacts of SSB consumption during pregnancy and infancy (Table 1). Warning label text was based on legislation pending in California at the time of message development [30].

2. Beverage sugar content imagery (Ix 2)

Participants randomized to Ix 2 received beverage-specific information on total sugar content for beverages (Table 1). Sugar content was based on nutrition facts labels of the sample beverages used in the images. Sugar quantity was illustrated in messages.

3. Attention control (AC)

AC participants received infant safety messages on topics such as infant immunizations and car safety (Table 1). Messages were based on HealthyChildren.org, a site published and maintained by the American Academy of Pediatrics [31].

### 2.4. Outcomes

The main outcome was change in habitual daily maternal SSB consumption over one month for each intervention arm compared to the attention control group. We defined SSBs as all beverages with added sugar: regular sodas, fruit/juice drinks, sport drinks (e.g., fluid or electrolyte replacement beverages), energy drinks, and other beverages that contain added caloric sweeteners such as flavored milks and sweetened teas or coffees [32]. Because 100% fruit juice (henceforth termed juice) does not contain added sugar, we did not include juice as an SSB. To measure maternal average daily beverage consumption, study staff administered the BEVQ-15, a validated and reliable quantitative 15-item beverage frequency questionnaire that can be administered in about 2 min with a 4th grade readability score [33]. Similar methodology has been reported as valid and reliable in a Hispanic/Latino population for measuring SSB consumption [34]. We used container samples or pictures of container samples to assist with maternal response for serving size information. We estimated habitual daily intake of SSBs in calories (kcals) using methodology previously described that includes use of food composition tables [33,35]. For the main outcome, we calculated the difference in change of maternal SSB consumption (kcal) from baseline to 1-month follow-up for each intervention arm compared to AC. 

In secondary outcomes, to assess whether interventions affected consumption of other types of beverages (i.e., substitution or displacement of non-SSBs), we used responses from the BEVQ-15 at baseline and follow-up to calculate maternal habitual daily intake of juice in calories (kcals), non-caloric artificially sweetened beverages (ASBs), water in volume (oz), and total beverage volume (oz) and energy (kcals).

For secondary outcomes of infant beverage consumption, the study staff used container samples to assist with administration of validated quantitative beverage frequency questionnaire for infants [36] and questions about breastmilk and formula use [37]. We also estimated infant daily consumption of SSBs, juice, cow’s milk, unsweetened coffee/tea, artificially sweetened/diet beverages, water, and other beverages to calculate total beverage ounces and kcals consumed daily. Given expert recommendations for infants to avoid added sugars before the age of 2 years [38,39], we classified infant SSB consumption dichotomously (any vs. none). For infants, we included formula and human (i.e., breast) milk consumption from a bottle/cup in total beverage calculations. 

For process measures, we asked questions about the frequency of reading mobile messages and viewing linked images (dose). We queried about satisfaction with the intervention.

For covariates, we collected information including study subject age, sex, race/ethnicity, pregnancy status, marital status, highest education level for self and partner (if applicable), household size, and household income. For the subset of parents with infants aged < 24 months, we obtained information on the child’s age, sex, race/ethnicity, and birth weight.

### 2.5. Sample Size

We estimated that 225 participants with complete baseline and follow-up visits would provide 98% power to detect a 30 kcal (16 oz of SSB weekly on average) reduction in average daily SSB consumption assuming equal standard deviations estimated at 30 kcal. To account for an estimated attrition rate of 25% over the 1-month intervention in keeping with other inventions in demographically similar patient populations [40], we estimated we would need to recruit up to 300 participants to meet target of 225 participants with complete data.

### 2.6. Randomization

Prior to Phase 1 recruitment, we stratified according to language (English, Spanish), pregnancy status (pregnant, non-pregnant), and recruitment site (two sites). For Phase 2 randomization, recruitment was conducted from a single site where all participants were non-pregnant with an infant and stratified according to language (English, Spanish) for eight strata in Phase 1 and two strata in Phase 2.

The study biostatistician generated the random allocation sequence using a computer algorithm. The study intervention coordinator concealed intervention allocation by coding the trial arms using neutral terminology and storing the code in a secure file. Intervention allocations were sealed in sequentially numbered envelopes. After completion of baseline data collection, study staff opened the sealed envelope and assigned the allocation. All study staff performing data collection and analysis were blinded to study arm allocation.

### 2.7. Statistical Analysis

#### 2.7.1. Primary Analysis

The main outcome was the 1-month change in maternal SSB consumption. Within each study arm, we examined change in maternal daily SSB calorie intake from baseline and 1-month using the Wilcoxon signed-rank test. To evaluate the effects of each of the two interventions compared to the AC, we completed two analyses. First, we used a weighted mean approach to estimate the average change in outcomes according to the treatment arm. Second, in linear regression analyses, we adjusted for blocking covariates used during randomization (language, pregnancy status, and site), and further adjusted for covariates with imbalance. 

#### 2.7.2. Sensitivity Analyses

In sensitivity analyses of the primary outcome, we examined the potential impact of participant exclusion due to loss to follow-up and outliers. First, to account for loss to follow-up, we used baseline information of participants without follow-up data to replicate the sample that would have been observed without attrition to estimate intention-to-treat effects. To achieve this, we used logistic regression to estimate individual propensities for attrition based on baseline characteristics. We then applied inverse probability weights in the multiple linear regression model to up-weight outcomes of complete cases whose baseline characteristics were similar to those with missing follow-up visits. In the second sensitivity analysis, we used Cook’s distance to identify potentially influential data points. After removing these outliers, we refitted the multiple linear regression model, and re-assessed the estimated treatment effects and 95% CI.

#### 2.7.3. Secondary Analyses

In secondary analyses, we used linear or logistic regression to examine intervention effects on changes in maternal and infant secondary beverage consumption outcomes. We adjusted for blocking covariates used in randomization (language, pregnancy status, and site) and baseline maternal/household covariates with imbalance (maternal age and household income). For infant outcomes, we additionally adjusted for infant covariates with imbalance (infant age and sex). For process measures, we used descriptive statistics to examine fidelity and satisfaction across intervention arms. 

All analyses were carried out in R Statistical Software (version 4.0.1). A priori significance level was 0.025 for two-tailed statistical tests after multiple testing adjustment for the primary outcome and 0.05 for secondary analyses.

## 3. Results

### 3.1. Study Participants

In Phase 1, we performed in-person recruitment of 110 families in the first 1000 days from two WIC sites from 27 January to 13 March 2020. Phase 1 follow-up visits (27 February 2020–15 April 2020) were done in person (17%), by computer-assisted telephone visit (73%), and by video (11%). In Phase 2 recruitment (13 October 2020–6 January 2021), all baseline visits were by video. For Phase 2 follow-up visits, 99% were by video. We ended Phase 2 recruitment when 225 participants completed study procedures. 

A total of 290 participants enrolled and 262 participants completed follow-up visits (Table 2, Figure S1). Similar proportions of participants completed follow-up visits across study arms.

Table 2 shows baseline characteristics of participants. All parents were female. Thus, we report results for maternal and infant outcomes. Most participants identified Spanish as their language of preference, 42% had annual household incomes under USD 20,000, and most participants were of Hispanic/Latino ethnicity. Most characteristics were similar across study arms, but we found differences in maternal age and household income (Table S1) according to study arm and site, as well as child age and sex among participants with an infant. 

### 3.2. Changes in Maternal SSB Consumption and Other Outcomes

#### 3.2.1. Primary Outcome

**Figure 1:**
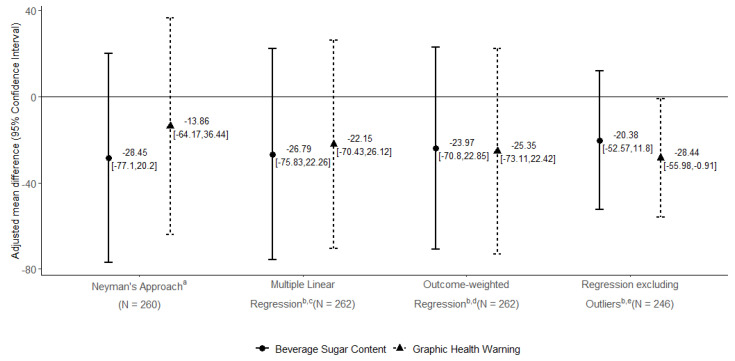
Primary and sensitivity analyses of the main outcome: adjusted difference in 1-month intervention effects on maternal SSB consumption. ^a^ Neyman’s approach usedpooled stratum-specific mean differences and variances based on our block randomization design (one stratum in Phase 1 only had two subjects assigned to the two intervention arms and no controls); ^b^ Adjusted for blocking covariates (site, pregnancy status, language), maternal age, and household income; ^c^ Linear regression analysis of mean change in maternal daily SSB kcal consumption; ^d^ Sensitivity analysis to account for missing data at follow-up; ^e^ Sensitivity analysis: multiple linear regression with influential points left out.

Baseline maternal mean daily SSB consumption was 163.92 kcal (SD 194.27) in Ix 1 (graphic health warnings), 158.93 kcal (SD 185.47) in Ix 2 (beverage sugar content), and 135.83 kcal (SD 192.26) in the AC (attention control). Maternal mean daily SSB consumption decreased significantly over 1 month in Ix 1 (−65.50 kcal, *p*-value < 0.0001), Ix 2 (−79.69 kcal, *p*-value < 0.0001), and AC (−45.81 kcal, *p*-value 0.007) groups (Table 3). In the primary analyses, compared to AC, the adjusted mean difference in 1-month change for maternal SSB consumption was not statistically significant for Ix 1 (−22.15 kcal (95% CI: −70.43, 26.12)) or Ix 2 (−26.79 kcal (95% CI: −75.83, 22.26)) (Figure 1).

In sensitivity analyses accounting for missing data at follow-up, the results were similar to the primary analysis. After excluding outliers, the effect size was greater in Ix 1 and confidence intervals narrowed in both Ix 1 (−28.44 kcal (95% CI: −55.98, −0.91)) and Ix 2 (−20.38 kcal (95% CI: −52.57, 11.80)) compared to AC over one month (Figure 1).

#### 3.2.2. Secondary Outcomes

In secondary analyses, we found decreases in maternal mean juice consumption over 1 month in Ix 1 (−82.41 kcal, *p*-value < 0.001), Ix 2 (−57.24 kcal, *p*-value < 0.002), and AC (−48.45 kcal, *p*-value 0.006) groups (Table S2). Maternal total energy intake from beverages decreased significantly for the Ix 1 (−155.41 kcal, *p*-value < 0.0001), Ix 2 (−177.61 kcal, *p*-value < 0.0001), and AC arms (−95.61 kcal, *p*-value 0.001). For total beverage volume intake, Ix 1 and Ix 2—but not the attention control arm—decreased mean consumption over 1 month. In adjusted models, we found no difference in 1-month change for any maternal beverage outcomes among intervention groups compared to AC.

For secondary outcomes of infant beverage consumption (Table S3), among the 238 infants with maternal report of beverage consumption at baseline and at 1 month, low prevalence of any SSB consumption was reported at baseline in Ix 1 (8.0%), Ix 1 (8.0%), and AC (10.3%) groups, and logistic regression models could not be fitted because of the small number of infants with any consumption. We did not find any statistically significant intervention effects for infant SSB/juice combined, juice, water, breastmilk in a bottle, or unflavored cow milk consumption. 

#### 3.2.3. Process Measures 

Most participants in all arms reported reading all messages (Figure S2). Click-through rates were similar across arms (data not shown). Almost all participants reported they agreed or strongly agreed that they had high satisfaction with the intervention (Figure S2). No harms were reported.

## 4. Discussion

In this randomized controlled trial of two different SSB reduction text messaging interventions—graphic health warnings and beverage sugar content information—delivered by mHealth for one month to low-income, predominantly Hispanic/Latina mothers in the first 1000 days, we found no difference in 1-month change in maternal SSB consumption among intervention groups versus an attention control group. In sensitivity analyses excluding outliers, the adjusted mean difference in the 1-month change for mean maternal SSB consumption for graphic health warnings was significantly lower than the AC group by 28 kcal. Despite widespread disruptions in relation to COVID-19, we found that recruitment, randomization, and delivery of messages using mHealth was feasible and well-received during a critical life course period in a population that is disproportionately burdened by obesity. Overall, our results support the acceptability and potential promise of including text messaging and graphic health warning labels to curb SSB consumption as part of a suite of policies and public health interventions to promote health equity starting early in life but suggest that the use of text messages alone will not substantially impact SSB consumption.

We found no intervention effects in our main and secondary analyses. Although maternal SSB consumption decreased significantly in both intervention arms, the AC group also reported declines in maternal SSB, juice, and total energy consumption over the 1-month intervention. Study participants were recruited from WIC or health care visits; thus, a plausible explanation for maternal reduction in SSB and juice consumption could be the receipt of counseling received at WIC or health care visits. While data are still limited on the effects of COVID-19 on specific health behaviors, another possible explanation for reduction in SSB consumption in all arms could be changes in food access and perceived need to be healthy related to the COVID-19 pandemic, which arrived in New York City after study recruitment began. Thus, related isolation measures or increased attention to health may have reduced SSB access and consumption in all arms. 

Our text messaging intervention was longer in duration and used a different messaging delivery modality than prior SSB messaging interventions. In the existing SSB messaging intervention, messages are tested in a virtual or real-world point-of-selection. In those studies, the main outcomes focus on responses or beverage selection and suggest that the use of warning labels shifts behaviors. In our study, we delivered text messages and examined average daily beverage consumption, rather than a single point-of-selection. Because we measured average daily consumption over 1 month, we were unable to study whether responses to messages changed over time, and whether there was an extinguishing effect. Additionally, although our intervention was longer in duration than some other similar studies, one month is still a relatively short intervention duration and may not be sufficient to change behaviors. Unidirectional text messaging may be helpful for increasing knowledge or shifting attitudes and intentions for behavior change. Future research using text messaging should examine different facets of behavior change frameworks to understand how the receipt of text messages may help support multi-component interventions. Existing research suggests that labels at the point-of-selection could be effective at changing beverage consumption [41,42,43,44,45,46,47]. However, widespread policy change in the U.S. is unlikely to result in systematic labelling of unhealthy beverage options. Thus, alternative modes of message delivery are needed to curb SSB consumption in high-risk groups. Our results suggest that unidirectional text messaging alone will not be sufficient to change SSB consumption but may be helpful in supporting broader interventions to curb SSB consumption.

Evidence supports effectiveness of health warnings on SSB and other unhealthy products [41,44,45]. Several states and cities in the U. S. have proposed legislation to require use of text-only warning labels on SSBs, but to date warning label laws have not been implemented [30,48]. Emerging data support the use of warning labels that, in addition to text, graphically depict evidence-based adverse health outcomes of SSB consumption [41,42,43,44,45,46,47]. Most prior research has focused on SSB purchasing, SSB selection, or other age groups. In a field study of adults in a hospital cafeteria, the use of graphic health warnings at the point of sale resulted in a reduction in SSB purchases [44]. In another field study, warning labels with an icon (triangle-exclamation mark) reduced SSB consumption by 14.5% in the intervention cafeteria relative to the control cafeterias [47]. A recent cross-sectional online RCT among parents of children aged 2–12 years found that graphic health warning labels and icon warning labels were perceived as more effective than text-only messages [45]. In our prior qualitative research, pregnant women and parents of infants identified graphic warning labels as the preferred option to motivate SSB reduction for themselves and their infants [24]. Similarly, a mixed-methods study found that young adults perceived warning labels with graphics or icons to be more effective and preferable to text-only labels [49].

The current study is the first experimental study conducted in a real-world setting to examine the effect of graphic health warning labels delivered by mHealth over a 1-month period on SSB consumption in a predominantly racial/ethnic minority, low-income, bilingual population in pregnancy and infancy. Our study adds to the literature by investigating the acceptability of graphic health warning labels among pregnant women and mothers of infants. The results of our sensitivity analyses suggest that exposure to graphic health warnings nudged maternal SSB consumption downward. In secondary analyses, we found decreases in maternal energy intake from juice and total beverages in the graphic health warning label group (Ix 1), but compared to changes in AC consumption, the findings were not statistically significant. Ix 1 had no changes in water or ASB consumption, suggesting that graphic health warnings did not lead to replacement with ASBs or other beverages. Although our results suggest that text messaging alone will not be sufficient to substantially reduce SSB consumption in this population, a growing evidence base suggests that graphic health warnings should be used by public health/mass media campaigns and other multi-pronged interventions to communicate the health risks of SSB consumption [41,42,43,44,45,46,47]. 

Although maternal SSB consumption declined in the beverage sugar content information group (Ix 2), the difference was not statistically significant compared to the AC group in any models. Recent changes to the nutrition facts labels in the U.S. include information about added sugar [50], but they have not yet required front-of-package labelling for food and beverages high in added sugar and other nutrients similar to those in other countries [51]. In our recent work, we found that families preferred illustrative depictions of sugar content in teaspoons or cubes [24]. The current results suggest that providing beverage sugar content information by mHealth is feasible and acceptable to pregnant women and mothers of infants.

A main limitation to this study is the potential for recall bias in relation to social desirability bias. We reduced this likelihood by using a valid and reliable quantitative beverage frequency questionnaire that has been used in other interventions [52]. Additionally, all parents were female and most were Hispanic/Latina, limiting the generalizability of results but providing information on a population that is disproportionately burdened by obesity. 

## 5. Conclusions

In this RCT, text messages with graphic beverage health warnings or beverage sugar content information delivered by mHealth for one month did not reduce maternal SSB consumption in the first 1000 days among predominantly low-income Hispanic/Latina women.

## Figures and Tables

**Table 1:** Sample messages in a three-arm randomized controlled trial of text messaging during pregnancy and infancy.

	Ix 1: Graphic Health Warnings	Ix 2: Sugar Content Messages	AC: Attention Control
Personalized Text Message	Hey [NAME], did you know that drinking sugary drinks can lead to health problems for you and your baby?	[NAME], What drink has as much sugar as four donuts? Click to see!	Hey [NAME], do you know that babies should always sleep on their backs? Click to learn more!
Linked Health Message	* 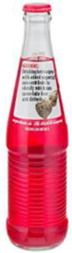 *	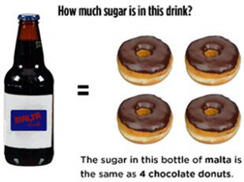	* 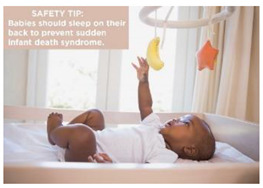 *

Samples for intervention 1 (Ix1), intervention 2 (Ix 2), and attention control (AC).

**Table 2:** Maternal, household, and child characteristics according to intervention condition. Data from 290 participants enrolled in a three-arm randomized controlled trial of healthy beverage messaging by mHealth during the first 1000 days.

	Overall	Ix 1: Graphic Health Warning	Ix 2: Beverage Sugar Content	AC: Attention Control
N	290	98	98	94
Maternal/Household Baseline Characteristics				
Maternal age, mean (SD), years	30.53 (6.30)	31.06 (6.30)	30.17 (6.11)	30.35 (6.53)
Pregnant participant, *n* (%)	28 (9.7)	11 (11.2)	10 (10.2)	7 (7.4)
Spanish language preference, *n* (%)	192 (66.2)	65 (66.3)	64 (65.3)	63 (67.0)
Annual household income, *n* (%)				
<USD 20,000/y	123 (42.4)	40 (40.8)	48 (49.0)	35 (37.2)
>USD 20,000/y	90 (31.0)	35 (35.7)	21 (21.4)	34 (36.2)
Do not Know	77 (26.6)	23 (23.5)	29 (29.6)	25 (26.6)
Maternal Race/ethnicity, *n* (%)				
Hispanic/Latina	265 (91.4)	89 (90.8)	88 (89.8)	88 (93.6)
White, non-Hispanic	2 (0.7)	0 (0.0)	1 (1.0)	1 (1.1)
Black, non-Hispanic	21 (7.2)	8 (8.2)	8 (8.2)	5 (5.3)
Current WIC enrollment, *n* (%)	272 (93.8)	90 (91.8)	95 (96.9)	87 (92.6)
N, Subset with an Infant	262	87	88	87
Infant Characteristic				
Female, *n* (%)	139 (53.1)	44 (50.6)	45 (51.1)	50 (57.5)
Age at baseline, mean (SD), years	0.67 (0.51)	0.70 (0.52)	0.64 (0.56)	0.66 (0.46)

USD: U.S. Dollars; WIC: Special Supplemental Nutrition Program for Women, Infants, and Children.

**Table 3:** Maternal sugar-sweetened beverage (SSB) consumption according to intervention condition.

	Main Outcome: Maternal Habitual Daily SSB Consumption (kcal)			
	Baseline	1 Month	1-Month Change	Within-Group *p*-Value ^a^
Intervention Condition	Mean (SD)	Mean (SD)	Mean Change (SD)	
Ix 1: Graphic Health Warning	163.92 (194.27)	98.43 (127.90)	−65.50 (184.79)	<0.0001
Ix 2: Beverage Sugar Content	158.93 (185.47)	79.24 (107.11)	−79.69 (171.31)	<0.0001
AC: Attention Control	135.83 (192.26)	90.03 (157.39)	−45.81 (135.12)	0.007

Data from 262 participants enrolled in a three-arm randomized controlled trial of healthy beverage messaging by mHealth during the first 1000 days. ^a^ Wilcoxon signed-rank test comparing maternal SSB consumption within each arm at baseline and follow-up.

## Data Availability

The data presented in this study are available on request from the corresponding author. The data are not publicly available due to privacy restrictions.

## Supplementary Materials

The following are available online at https://www.mdpi.com/article/10.3390/nu13124367/s1, Figure S1: CONSORT flow diagram for three-arm randomized controlled trial of healthy beverage messaging by mHealth during pregnancy and infancy (two intervention arms and one attention control), Figure S2: Participants’ self-report of intervention satisfaction and fidelity. Table S1: Parent and child characteristics according to stratified randomization. Table S2: Secondary outcomes: maternal beverage consumption according to the intervention arm. Data from 262 participants with completed follow-up visits. Table S3: Infant beverage consumption according to intervention arm. Data from 238 infants with completed follow-up visits.
