# Supplementary material for: Text Messages to Curb Sugar-Sweetened Beverage Consumption among Pregnant Women and Mothers: A Mobile Health Randomized Controlled Trial
Source: Nutrients. 2021 Dec 5;13(12):4367. doi: 10.3390/nu13124367 (PMC8703966; doi:10.3390/nu13124367)

Figure S1. CONSORT Flow Diagram for 3-Arm Randomized Controlled Trial of Healthy Beverage Messaging by mHealth During Pregnancy and Infancy (2 Intervention Arms, 1 Attention Control).

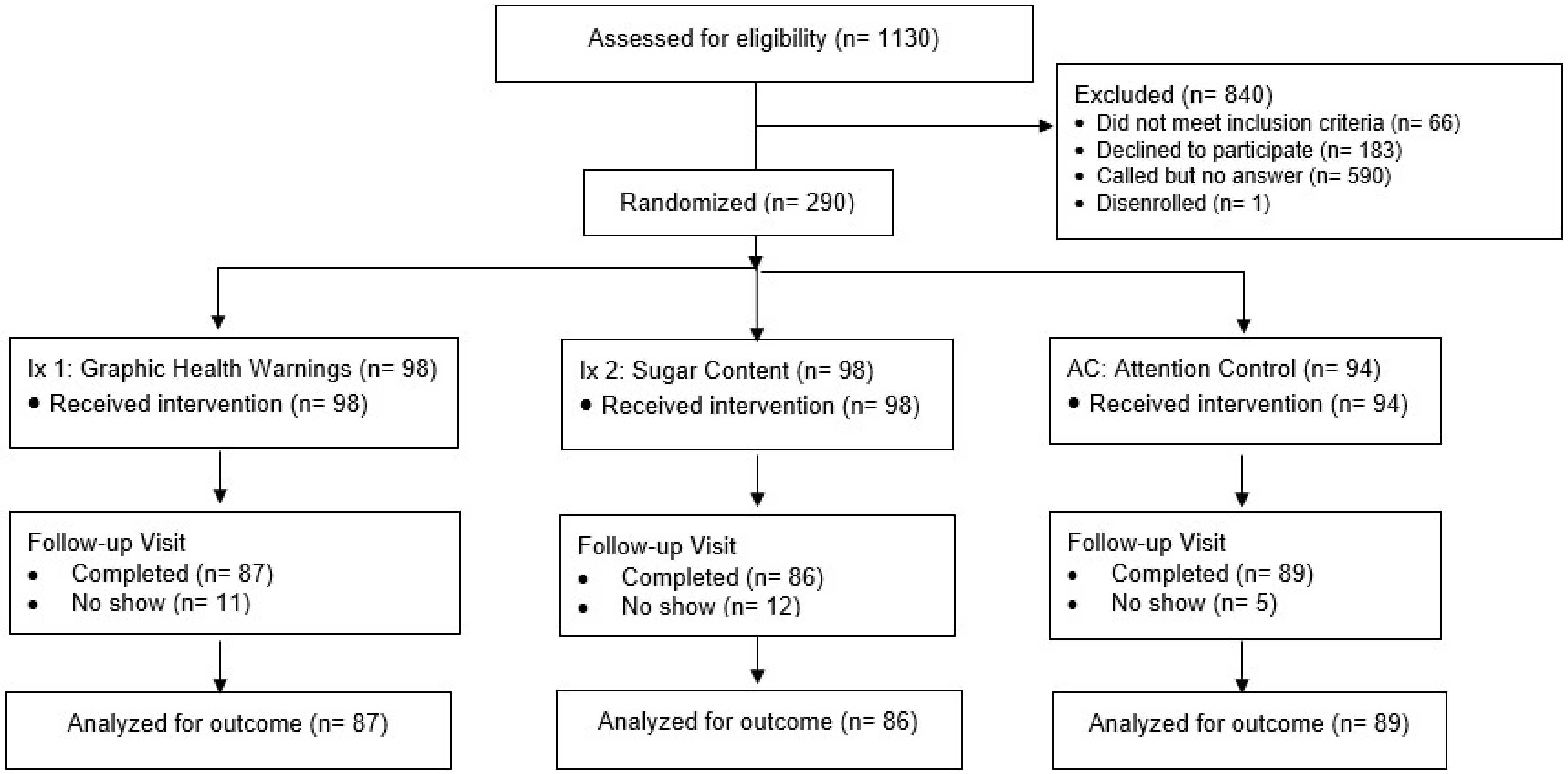

Table S1. Parent and Child Characteristics According to Stratified Randomization. Data from 290 Participants Enrolled in a 3-Arm Randomized Controlled Trial of Healthy Beverage Messaging by mHealth During Pregnancy and Infancy

	**Phase 1 Recruitment**						**Phase 2 Recruitment**		
	**Site 1 (N= 79)**			**Site 2 (N= 31)**			**Site 3 (N= 180)**		
	Ix 1: Warning	Ix 2: Sugar	AC: Control	Ix 1: Warning	Ix 2: Sugar	AC: Control	Ix 1: Warning	Ix 2: Sugar	AC: Control
**N**	N= 27	N= 25	N= 27	N= 11	N= 13	N= 7	N= 60	N= 60	N= 60
**Family/Household characteristic**									
Maternal age, mean (SD), y	29.38 (6.51)	28.16 (6.66)	29.12 (6.02)	27.81 (6.99)	28.39 (6.93)	28.91 (9.20)	32.40 (5.77)	31.39 (5.44)	31.06 (6.42)
Pregnancy status									
Pregnant, n (%)	7 (25.9)	5 (20.0)	5 (18.5)	4 (36.4)	5 (38.5)	2 (28.6)	0 (0.0)	0 (0.0)	0 (0.0)
Non-Pregnant, n (%)	20 (74.1)	20 (80.0)	22 (81.5)	7 (63.6)	8 (61.5)	5 (71.4)	60 (100.0)	60 (100.0)	60 (100.0)
Language preference, n (%)									
Spanish	16 (59.3)	15 (60.0)	16 (59.3)	7 (63.6)	7 (53.8)	5 (71.4)	42 (70.0)	42 (70.0)	42 (70.0)
English	11 (40.7)	10 (40.0)	11 (40.7)	4 (36.4)	6 (46.2)	2 (28.6)	18 (30.0)	18 (30.0)	18 (30.0)
Education, any college, n (%)	14 (51.9)	13 (54.2)	14 (51.9)	7 (63.6)	6 (46.2)	1 (14.3)	40 (66.7)	32 (53.3)	38 (64.4)
Annual household income, n (%)									
Don’t know	5 (18.5)	7 (28.0)	5 (18.5)	6 (54.5)	7 (53.8)	5 (71.4)	12 (20.0)	15 (25.0)	15 (25.0)
≤$20,000/y	13 (48.1)	12 (48.0)	8 (29.6)	3 (27.3)	5 (38.5)	2 (28.6)	24 (40.0)	31 (51.7)	25 (41.7)
>$20,000/y	9 (33.3)	6 (24.0)	14 (51.9)	2 (18.2)	1 (7.7)	0 (0.0)	24 (40.0)	14 (23.3)	20 (33.3)

Table S1. Cont

Maternal Race/ethnicity, n (%)									
Hispanic	25 (92.6)	23 (92.0)	27 (100.0)	11 (100.0)	12 (92.3)	6 (85.7)	53 (88.3)	53 (88.3)	55 (91.7)
White, non-Hispanic	0 (0.0)	0 (0.0)	0 (0.0)	0 (0.0)	0 (0.0)	0 (0.0)	0 (0.0)	1 (1.7)	1 (1.7)
Black, non-Hispanic	2 (7.4)	2 (8.0)	0 (0.0)	0 (0.0)	1 (7.7)	1 (14.3)	6 (10.0)	5 (8.3)	4 (6.7)
Other/More than 1 race	0 (0.0)	0 (0.0)	0 (0.0)	0 (0.0)	0 (0.0)	0 (0.0)	1 (1.7)	0 (0.0)	0 (0.0)
Maternal pre-pregnancy BMI, mean (SD), kg/m2	27.66 (6.48)	25.77 (4.61)	26.68 (4.39)	27.53 (5.06)	29.62 (7.27)	28.97 (10.07)	26.74 (5.92)	27.68 (5.75)	28.92 (6.89)
**Infant**		**Site 1 (N= 62)**			**Site 2 (N= 20)**			**Site 3 (N= 180)**	
	Ix 1: Warning	Ix 2: Sugar	AC: Control	Ix 1: Warning	Ix 2: Sugar	AC: Control	Ix 1: Warning	Ix 2: Sugar	AC: Control
	N= 20	N= 20	N= 22	N= 7	N= 8	N= 5	N= 60	N= 60	N= 60
Age at baseline, mean (SD), years	0.57 (0.48)	0.50 (0.59)	0.58 (0.48)	0.37 (0.30)	0.43 (0.30)	0.72 (0.58)	0.78 (0.53)	0.72 (0.56)	0.69 (0.46)
Female, n (%)	12 (60.0)	11 (55.0)	10 (45.5)	3 (42.9)	3 (37.5)	2 (40.0)	29 (48.3)	31 (51.7)	38 (63.3)

Table S2. Secondary Outcomes: Maternal Beverage Consumption According to Intervention Arm. Data from 262 Participants with Completed Follow-up Visits.

	**Secondary Outcomes: Maternal Beverage Consumption**				
	**Baseline**	**1 month**	**1-Month Change**		
	Mean (SD)	Mean (SD)	Mean Change (SD)	Within-group p-value[a]	Adjusted Mean Difference in Change (95% CI)[b]
Maternal juice intake, kcal					
Ix 1: Graphic Health Warning	162.53 (213.82)	80.12 (109.89)	-82.41 (178.04)	<0.0001	-32.99 (-81.62, 15.63)
Ix 2: Beverage Sugar Content	138.76 (181.46)	81.52 (128.72)	-57.24 (143.62)	0.0002	0.48 (-48.92, 49.88)
AC: Attention Control	122.62 (169.76)	74.17 (114.21)	-48.45 (182.25)	0.006	0.00 (Ref)
Maternal water intake, oz					
Ix 1: Graphic Health Warning	35.34 (17.72)	35.25 (16.19)	-0.08 (21.82)	0.96	-0.28 (-6.00, 5.44)
Ix 2: Beverage Sugar Content	35.73 (17.24)	34.71 (16.88)	-1.02 (17.25)	0.53	-0.94 (-6.75, 4.87)
AC: Attention Control	32.82 (16.91)	33.65 (16.70)	0.82 (17.62)	0.79	0.00 (Ref)
Maternal artificially-sweetened drink intake, oz					
Ix 1: Graphic Health Warning	0.48 (3.88)	0.23 (1.54)	-0.25 (2.76)	0.58	-0.27 (-1.02, 0.48)
Ix 2: Beverage Sugar Content	0.39 (2.09)	0.07 (0.62)	-0.32 (2.19)	0.25	-0.23 (-0.99, 0.53)
AC: Attention Control	0.27 (2.54)	0.28 (1.45)	0.01(2.53)	0.59	0.00 (Ref)

Table S2. Cont

Maternal energy intake from beverages, kcal					
Ix 1: Graphic Health Warning	420.05 (350.32)	264.64 (199.08)	-155.41 (295.82)	<0.0001	-58.66 (-147.59, 30.27)
Ix 2: Beverage Sugar Content	408.11 (430.40)	230.50 (219.94)	-177.61 (351.80)	<0.0001	-60.46 (-150.82, 29.89)
AC: Attention Control	331.46 (317.40)	235.85 (230.48)	-95.61 (275.32)	0.001	0.00 (Ref)
Maternal total beverage volume intake, oz					
Ix 1: Graphic Health Warning	66.58 (30.41)	55.75 (19.11)	-10.83 (30.79)	0.007	-5.25 (-13.73, 3.22)
Ix 2: Beverage Sugar Content	65.87 (30.06)	52.66 (23.47)	-13.21 (29.20)	<0.0001	-5.94 (-14.55, 2.66)
AC: Attention Control	57.43 (26.93)	52.42 (24.71)	-5.02 (26.91)	0.13	0.00 (Ref)

[a] Wilcoxon signed-rank test comparing other maternal beverages consumption within each arm at baseline and follow-up

[b] Multiple linear regressions adjusted for blocking covariates (site, pregnancy status, language), maternal age and household income

Table S3. Infant Beverage Consumption According to Intervention Arm. Data from 238 Infants with Completed Follow-up Visits.

	Infant Beverage Consumption		
	**Baseline**	**1 month**	
**Binary outcome**	**n (%)**	**n (%)**	**Adjusted Odds Ratio (95% CI)[a]**
Infant SSB and 100% juice intake, Any			
Ix 1: Graphic health warning	24 (10.1)	21 (8.8)	0.48 (0.20, 1.10)
Ix 2: Beverage sugar content	22 (9.2)	23 (9.7)	0.65 (0.27, 1.52)
AC: Attention control	27 (11.3)	29 (12.2)	1.00 (Ref)
**Continuous outcomes**	**Mean (SD)**	**Mean (SD)**	**Adjusted Mean Difference in Change (95% CI)[b]**
Infant juice intake, oz			
Ix 1: Graphic health warning	1.13 (2.72)	0.80 (1.77)	-0.90 (-1.95, 0.16)
Ix 2: Beverage sugar content	1.55 (3.84)	1.83 (4.11)	-0.43 (-1.50, 0.64)
AC: Attention control	1.01 (2.36)	1.59 (4.67)	Ref
Infant water intake, oz			
Ix 1: Graphic health warning	4.13 (7.8)	4.50 (7.11)	0.48 (-1.91, 2.87)
Ix 2: Beverage sugar content	7.29 (11.77)	5.93 (8.69)	-0.96 (-3.38, 1.47)
AC: Attention control	5.45 (9.34)	5.51 (7.72)	Ref

Table S3. Cont

Infant breastmilk intake, oz			
Ix 1: Graphic health warning	1.58 (5.62)	1.30 (5.54)	-0.17 (-1.63, 1.28)
Ix 2: Beverage sugar content	0.72 (2.80)	1.67 (6.80)	0.77 (-0.71, 2.24)
AC: Attention control	0.41 (1.54)	0.61 (2.01)	Ref
Infant unflavored cow milk intake, oz			
Ix 1: Graphic health warning	3.31 (7.96)	4.35 (9.55)	-1.02 (-3.63, 1.58)
Ix 2: Beverage Sugar Content	4.40 (9.89)	4.25 (9.56)	-1.89 (-4.53, 0.75)
AC: Attention control	3.31 (7.96)	4.35 (9.55)	Ref

[a] Multiple logistic regression adjusted for blocking covariates (site, pregnancy status, language); maternal age; household income; and infant age and sex.

[b] Multiple linear regression adjusted for blocking covariates (site, pregnancy status, language); maternal age; household income; and infant age and sex.

Figure S2. Participants' Self-report of Intervention Satisfaction and Fidelity. Data from 262 Participants at 1-month Follow-up.

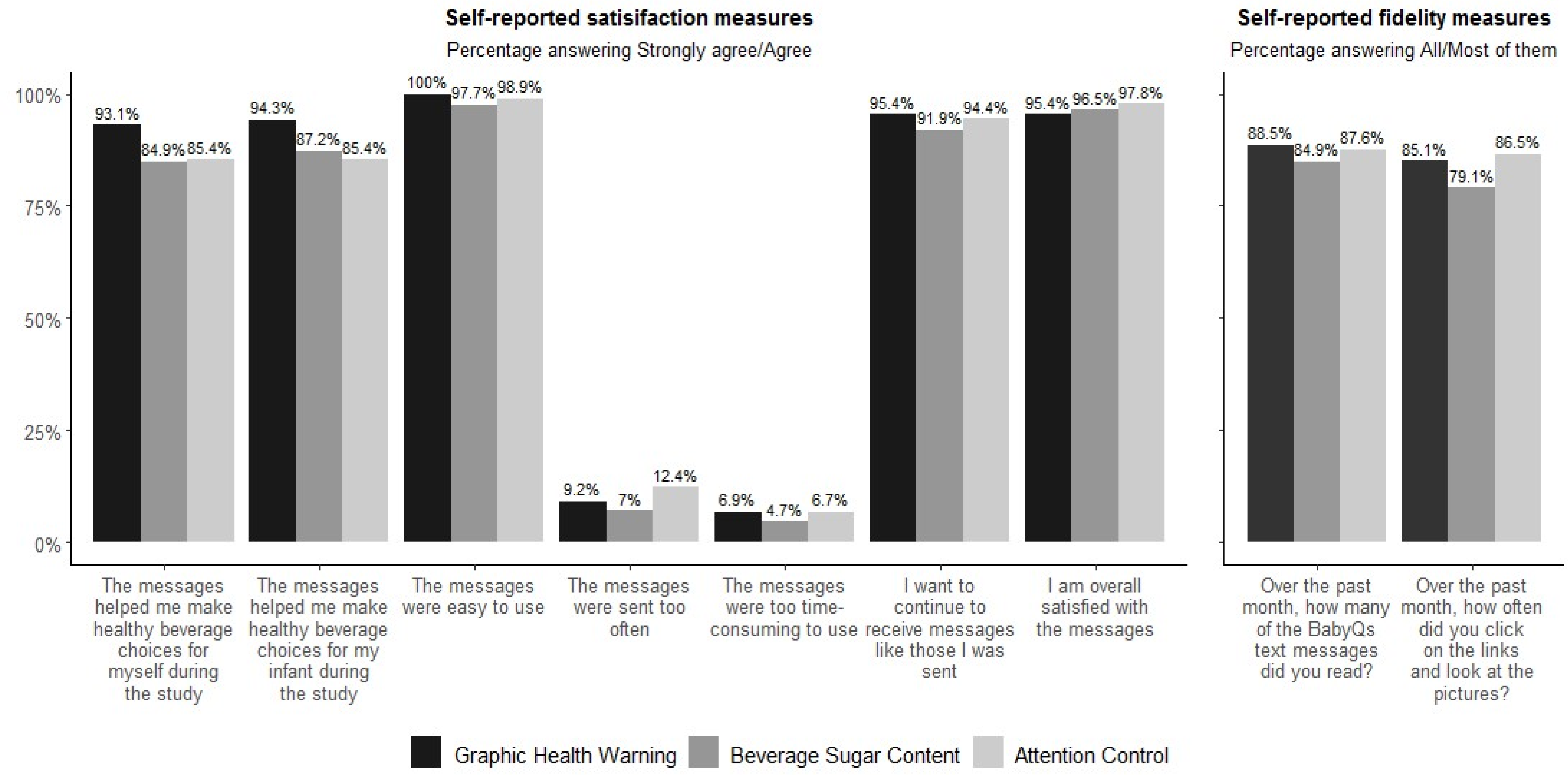

Supplement: Supplementary file 1 [file nutrients-13-04367-s001.zip › nutrients-1491864-supplementary.pdf]
